# Development of Enzymatic Recombinase Amplification Assays for the Rapid Visual Detection of HPV16/18

**DOI:** 10.4014/jmb.2304.04009

**Published:** 2023-05-19

**Authors:** Ning Ding, Wanwan Qi, Zihan Wu, Yaqin Zhang, Ruowei Xu, Qiannan Lin, Jin Zhu, Huilin Zhang

**Affiliations:** 1 Department of Obstetrics and Gynecology, Women's Hospital of Nanjing Medical University, Nanjing Maternity and Child Health Care Hospital, Nanjing 210004, P.R. China; 2 Centre for Diseases Prevention and Control of Eastern Theater, Nanjing 210018, P.R. China; 3 Department of Infectious Disease, Jiangsu Province Hospital and Nanjing Medical University First Affiliated Hospital, Nanjing 210029, P.R. China; 4 Nanjing Normal University, Nanjing 210023, P.R. China; 5 Changzhou Maternal and Child Health Care Hospital, Changzhou Medical Center, Nanjing Medical University, Changzhou 213004, P.R. China

**Keywords:** Enzymatic recombinase amplification, lateral flow dipstick, human papillomavirus, rapid visual detection

## Abstract

Human papillomavirus (HPV) types 16 and 18 are the major causes of cervical lesions and are associated with 71% of cervical cancer cases globally. However, public health infrastructures to support cervical cancer screening may be unavailable to women in low-resource areas. Therefore, sensitive, convenient, and cost-efficient diagnostic methods are required for the detection of HPV16/18. Here, we designed two novel methods, real-time ERA and ERA-LFD, based on enzymatic recombinase amplification (ERA) for quick point-of-care identification of the HPV E6/E7 genes. The entire detection process could be completed within 25 min at a constant low temperature (35–43°C), and the results of the combined methods could be present as the amplification curves or the bands presented on dipsticks and directly interpreted with the naked eye. The ERA assays evaluated using standard plasmids carrying the E6/E7 genes and clinical samples exhibited excellent specificity, as no cross-reaction with other common HPV types was observed. The detection limits of our ERA assays were 10^0^ and 10^1^ copies/μl for HPV16 and 18 respectively, which were comparable to those of the real-time PCR assay. Assessment of the clinical performance of the ERA assays using 114 cervical tissue samples demonstrated that they are highly consistent with real-time PCR, the gold standard for HPV detection. This study demonstrated that ERA-based assays possess excellent sensitivity, specificity, and repeatability for HPV16 and HPV18 detection with great potential to become robust diagnostic tools in local hospitals and field studies.

## Introduction

Cervical cancer (CC), which rank fourth among the most common cancer in women, continued to be a great threat to female health with an estimated 0.6 million new cases and 0.3 million deaths globally in the year 2020 [1]. It is well recognized that high-risk human papillomavirus (HR-HPV) infection is the major cause of CC and among 15 identified HR-HPV genotypes, HPV16 and 18 are the most frequent oncogenic types with high aggressiveness and persistence [2-4]. Due to over 70% of cervical cancer cases being associated with HPV16 and 18 [5], CC screening programs prioritize the detection of these two subtypes. With the popularization of CC screening programs and HPV vaccination programs, the mortality and morbidity of CC have significantly declined in many developed countries [6, 7]. However, some low/middle-income countries (LMICs) still lack effective screening programs, and approximately 88% of CC cases in 2020 occurred in these areas [8, 9]. Therefore, the development of an accurate, convenient, and cost-effective diagnostic method for detecting HPV16/18 in low-resource settings will be of great importance for preventing cervical lesions.

To date, there are more than 250 HPV test kits available in the global market, but most of them still need further experiments and clinical validation [10-12]. Hybrid capture 2 (HC2) and polymerase chain reaction (PCR) are two detection methods that are currently used most commonly among the tests approved by the U.S. Food and Drug Administration (FDA) for HPV primary screening. Both tests have high sensitivity and can be used as the gold standard in clinical practice [13]. HC2 assay can qualitatively detect 13 types of HPV, but it cannot identify specific subtypes and its specificity is relatively lower because of the cross-hybridization with multiple other HPV types [14]. Although PCR-based tests have the advantages of high sensitivity, specificity, and reproducibility, the requirement of expensive instruments and well-trained technicians, which is difficult to meet in resource-poor settings, limits its application in point-of-care testing (POCT) [15, 16].

Currently, several isothermal amplification techniques, such as loop-mediated isothermal amplification (LAMP) [17], recombinase-aided amplification (RAA) [18], and recombinase polymerase amplification (RPA)[19] have been developed for rapid nucleic acid detection. These new detection methods are featured by short consuming time and low-temperature demand and are quite suitable for field detection. Recently, an improved version of the RPA assay [20], the enzymatic recombinase amplification (ERA) assay was successfully applied to detect diverse pathogens with high sensitivity and specificity [21-24], and so far, there is no research on identifying HPV genotypes using ERA assay. The principle of ERA assay combines isothermal recombinase-driven targeted amplification with strand-displacement DNA synthesis [25]. In the amplification process, recombinase and accessory proteins can bind specific primers to form a recombinant complex. With the help of single-stranded DNA binding protein (SSB), the duplex DNA can be converted to single strands for specifically combining with the recombinant complex. Then, under the function of strand-displacing DNA polymerase, new complementary DNA strands can be exponentially amplified for further identification [23]. The ERA technology does not require any sophisticated equipment and can be performed at a constant temperature (optimally around 35-43°C) using a simple and portable heating device or water bath, which can be easily acquired at a low cost.

**Fig. 1:**
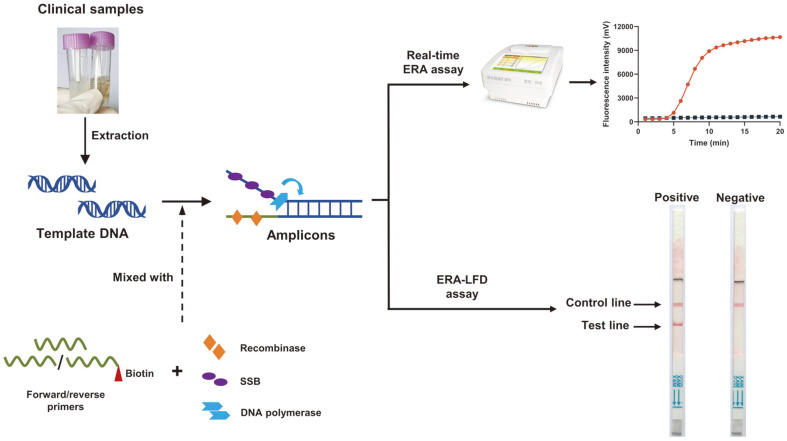
Schematic diagram of real-time ERA and ERA-LFD assays.

The amplification products of ERA assays can be detected in several ways, such as agarose gel electrophoresis, real-time fluorescence, and lateral flow dipsticks (LFD). Agarose gel electrophoresis can be applied to analyze the length and amounts of amplicons, but it takes a relatively long reaction time and requires other equipment [26]. In contrast, the fluorescent probe can monitor and analyze the results in real-time [27-29], while LFD is extremely portable and easy to operate and can read the results within 5min by the naked eye [30-32]. The probes and enzymes applied for the above methods are quite different. The exo-probe, which is used for real-time ERA assay, obtains a tetrahydrofuran (THF) between the fluorophore and quencher. The THF can be identified and split by exonuclease III and the separation of fluorophore and quencher will generate fluorescence signals [28, 29]. For ERA-LFD assay, endonuclease IV can identify the THF of the nfo-probe and convert the probe to a forward primer carrying a fluorophore at the 5¢ end [30]. With the function of a biotin-labeled backward primer, the amplicons will contain two antigenic markers at both ends. One of the markers can be captured by the test line of LFD and the other will be combined with a gold-labeled antibody. The product of ERA-LFD assay will finally be presented as the bands on the test line [31, 32]. The amplification process of the ERA technique is shown in Fig. 1.

In this study, we developed and validated two ERA-based assays-real-time ERA and ERA combined with LFD (ERA-LFD)—for the rapid and on-site detection of HPV 16 and 18. The assays can identify specific HPV types by targeting E6 and E7 genes, which encode oncoproteins that play a key role in the malignant process of cervical tissue [33]. Both methods can be completed within 25 min at 39°C and the results are presented as either fluorescence signals or as bands on the dipsticks, which can be directly observed with the naked eye.

## Materials and Methods

### Preparation of Standard Plasmids

The full-length E6 and E7 genes of HPV16/18 (GenBank Accession No. K02718.1 and No. AY262282.1) were synthesized and cloned into the pUC57 vector by Nanjing GenScript Biotech Co., Ltd. (China). The recombinant plasmids were quantified using a NanoDrop One spectrophotometer (Thermo Fisher Scientific, USA) and diluted 10 folds in Tris-EDTA buffer solution to acquire a series of concentrations ranging from 10^7^ to 10^0^ copies/μl, which can be used for evaluating the limits of detection of ERA assays. We also prepared plasmids containing the E6 and E7 genes of HPV31, 45, 66, and 73 for the specificity test (Table 1). All the plasmid solutions were stored at −20°C until use.

### Design and Screening of ERA Primer Sets

Information on the E6 and E7 open reading frames (ORFs) of HPV16 and HPV18 was obtained from the National Center for Biotechnology Information (NCBI). ERA primers were designed within the above regions using the Primer 6.0 software. Several design principles should be obeyed: the optimal length of the primers is typically 30 to 35 nucleotides long, and amplicon length is expected to be < 500 bp; the 5¢-ends of the reverse primers used for the ERA-LFD assay should be tabbed with biotin; primer dimers and hairpins should be avoided.

We designed three forward and three reverse candidate primers for HPV16 and 18, respectively. To screen for the optimal primer pair, a basic nucleic acid amplification kit (GenDx, China) was first employed and the reaction product was evaluated by agarose gel electrophoresis (AGE). However, the AGE assay is not sufficiently accurate and it is difficult to make a choice from similar results. Given this limitation, we established a real-time ERA assay for selection, whereby the fluorescence signal can be monitored during the reaction procedure and the results can be directedly read after the amplification procedure, which requires more than 30 min by AGE. Each primer set was evaluated by real-time ERA assay for three times and the optimal combination was selected through comparing the amplification curve and final fluorescence intensities. All the primers used in this study were synthesized by Sangon Biotech (China), and their sequence is shown in Table S1. The optimal primer pairs used in the following study were listed in Table 2.

### Design and Synthesis of ERA Probes

In this study, we design two types of DNA probes - the nfo-probe and exo-probe - which have the same sequence but different structures for ERA-LFD and real-time ERA assays. The nfo-probe should be controlled within 46 to 52 bp, which carries a 5-Carboxyfluorescein (FAM) at the 5¢ end, blocked with a polymerase extension blocking group (C3-spacer) at the 3¢ end, and tetrahydrofuran (THF) replaces the base located between 31 and 37 bp. While for the exo-probe, the FAM fluorophore was moved from the 5¢ ends to a thymine (T) base at 30 bases from the 5¢end, and the T base 15 bases from the 3¢ end was labeled by quencher Q(BHQ-1). A THF was inserted between FAM and BHQ-1 to substitute for one base. All the probes were synthesized by Sangon Biotech (China) and purified by high-performance liquid chromatography (HPLC). The sequence of nfo-probes and exo-probes were shown in Table 2.

### Establishment and Optimization of ERA Assays

The reaction composition of real-time ERA and ERA-LFD assays contained 10 μl of deliquescent agent, 2.4 μl of 10 μM ERA forward or reverse primer, 0.8 μl of 10 μM fluorescent probe, 2–8 μl of DNA sample, and RNase-free H2O added to 48 μl; negative control was the same set. The above components were fully mixed and added to PCR reaction tubes containing dried enzyme pellets; lastly, 2 μl of magnesium acetate was added to the caps of the PCR tubes, before closing the caps and vortex for 3 to 5 s. The reaction tubes were simultaneously spun and placed into a heating device to ensure the reaction was performed at 39°C for 20 min. The fluorescence curve of real-time ERA could be directly read using a portable amplification instrument, which can collect fluorescence signals every 30 sec. For ERA-LFD assay, the amplification product was diluted with RNase-free H2O for a ratio of 1:80 and transferred into a 1.5-mL centrifuge tube. The diluent was tested using LFD. The bottom part of the strip was immersed in the diluted solution. After incubating for 2 min at room temperature, we directly read the results with the naked eye from the test (T line) and the control lines (C line) on the strips. The presence of a T and C red line indicates a positive result; the absence of a red T line indicates a negative result or a result below the detection limit of the method. Normally, the red C line should appear after the reaction; if the C line is not present, the result is not credible (Fig. 1). The related reagents and thermal equipment were bought from GenDx Biotech Co., Ltd. (China).

To determine the optimal reaction conditions, we used real-time ERA assay to evaluate the optimal reaction temperature and the concentration of the primers. The reaction was performed at a series of temperature conditions: 35°C, 37°C, 39°C, 41°C, and 43°C. And five different final concentrations of primers were used for real-time ERA assay: 300, 360, 420, 480, and 540 nM. All the above experiments were performed in triplicate. Then, we chose ERA-LFD assay for the optimization of reaction time, and the amplification process was separately stopped at 5, 10, 15, 20, 25, and 30 min. Plasmids with high concentration (10^5^ copies/μl) and low concentration (10^1^ copies/μl) were used as templates and detected three times. After the reaction, the strips were placed in diluted amplification solution for 2 min and the bands on the test lines were compared.

### The Limit of Detection test of ERA Assays

A ten-fold serial dilution of HPV16-E6 and HPV18-E7 containing plasmids, varying from 10^7^ to 10^0^ copies/μl, were used as templates for real-time ERA and ERA-LFD assays under the previously verified optimal conditions. To further evaluate the sensitivity of ERA assays, we compare the performance of these two methods with real-time PCR assay [34], which is determined as the gold standard for the detection of HPV. The real-time PCR assay was performed according to the manufacturer protocol with a TB Green Premix Ex Taq II kit (Takara Bio., China) in a 20 μl volume containing 10 μl of TB Green Premix Ex Taq II (2X), 0.5 μl of forward or backward primers and 2 μl of templates. An Applied Biosystems Quant Studio 5 system (ThermoFisher Scientific) was applied for real-time PCR and the program was set as follows: 95°C for 5 min, followed by 40 cycles of 95°C for 10 s, 60°C for 20 s. The sequence of primers used in real-time PCR assay were shown in Table 2. All the above reactions were performed in triplicate and the limit of detection (LOD) was defined as the lowest concentration which can be detected in all three replicates.

### The Specificity Test of ERA Assays

To evaluate the specificity of the assays, plasmids containing E6 and E7 genes of 6 kinds of HR-HPV genotypes, including HPV-16, 18, 31, 45, 66, 73, were adopted for real-time ERA and ERA-LFD assays at the same time. Except for the synthetic plasmids, DNA templates of HPV33, 35, 39, 52, and 58 were extracted from clinical samples validated and distinguished by real-time PCR (Table 1). The amplification reaction was performed three times under the optimal conditions verified previously.

### Application of the ERA Assays in Clinical Samples

Cervical samples from 114 adult women (18–65 of age), including 36 HPV16 positive, 18 HPV18 positive, and 60 negative controls (14 patients positive for other HR-HPVs and 46 healthy individuals) were collected from Nanjing Maternal and Child Health Hospital in 2022. All samples were analyzed and diagnosed using real-time quantitative PCR. The entire procedure was approved by the Medical Ethics Committee of Nanjing Maternal and Child Health Care Hospital (2022KY-006). DNA was extracted from clinical samples using a viral RNA/DNA extraction kit (Takara Bio.) according to the manufacturer's protocol. The DNA extracts were stored at −20°C until further use.

## Results

### Screening of the Optimal Primer Combination for ERA Assays

**Fig. 2:**
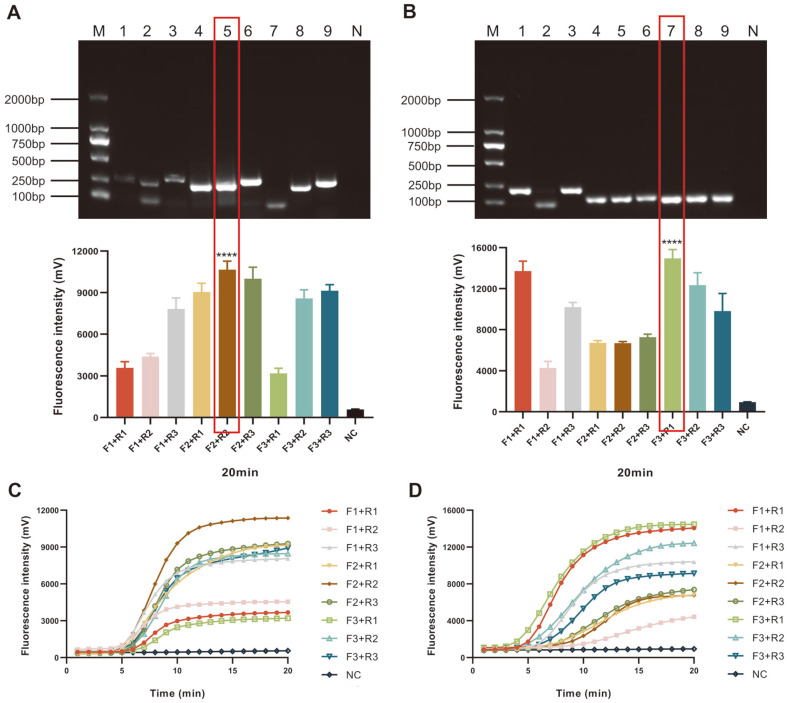
Screening for the optimal primer combinations for HPV16 and 18. (**A, B**) 1%AGE of ERA amplified products with nine different primer combinations of HPV16 and 18 and the final fluorescence intensities of real-time ERA assays using each primer set. Fluorescence curves of real-time ERA assays generated by 9 primer combinations of HPV16 (**C**) and 18 (**D**). M: DL 2000 DNA marker; N/NC: negative control; 1-9: F1+R1, F1+R2, F1+R3, F2+R1, F2+R2, F2+R3, F3+R1, F3+R2, F3+R3; ****: *p* < 0.0001.

As mentioned above, we designed probes within the conservative E6 and E7 regions of HPV16 and 18. Next, we selected three upstream and three downstream candidate primers surrounding the probe. All the candidate primers are shown in Table S1. First, we evaluated the amplification efficiency of the nine primer combinations using 1% AGE (Figs. 2A and 2B). However, AGE cannot precisely reflect the amplicons; for example, the bands of the F2/R1 and F2/R2 of HPV16 have similar brightness, which renders direct selection challenging. Moreover, the process of AGE requires more than 30 min and, during which, opening the reaction tubes may cause aerosol pollution and affect the accuracy of results.

Compared with this method, using real-time ERA assay can remedy the above shortcomings. As shown in Fig. 2, when using the F2/R2 combination of HPV16 and F3/R1 of HPV18, the strongest amplification signal was observed when detecting 10^5^ copies of plasmid DNA with an amplification time of 20 min. The optimal combination of candidate primers was visually recognized and applied in subsequent experiments (Table 2).

### Optimization of Reaction Conditions for ERA Assays

**Fig. 3:**
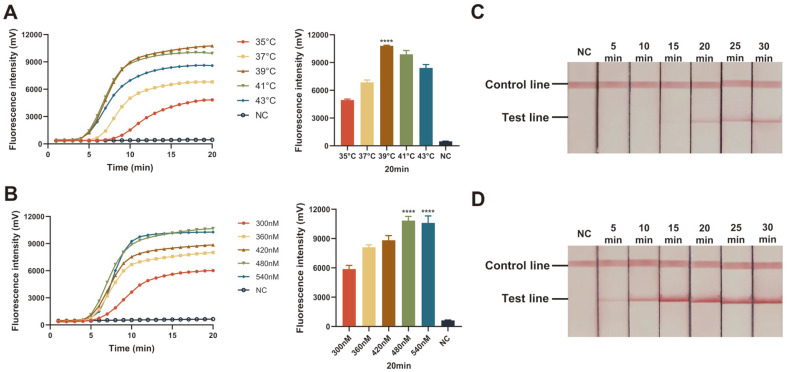
Optimization of ERA reaction conditions for the detection of pHPV-16-E6/7. (**A**) Fluorescence curves and final fluorescence intensities of real-time ERA assay performed at a broad range of amplification temperatures (35°–43°C) to analyze the optimal reaction temperature. (**B**) Fluorescence curves and final fluorescence values of real-time ERA assay performed with different concentrations of primers (300, 360, 420, 480, and 540 nM) to evaluate the optimal concentration of primer sets. ERA-LFD assay was conducted with 10^1^ copies/μl (**C**) and 10^5^ copies/μl (**D**) of HPV16-E6/E7 plasmids to evaluate the optimal reaction time. NC: negative control; ****: *p* < 0.0001.

To determine the optimal reaction temperature, real-time ERA assay was performed at a series of temperatures (35, 37, 39, 41, and 43°C), and the best amplification curve and highest fluorescence value were observed at 39°C (Fig. 3A); Thus, we finally chose 39°C for the follow-up experiments. We further tested various concentrations of the primer (300, 360, 420, 480, and 540 nM) using real-time ERA assay. The results show that when the primer concentration exceeded 480 nM, the final fluorescence value no longer increased (Fig. 3B).

Subsequently, we performed the ERA-LFD assay for different reaction times, ranging from 5 to 30 min, with 10^5^ and 10^1^ copies/μl of HPV16-E6/E7 plasmids, respectively. When we used high-concentration plasmid as the template, the band of the test line became visible after 5 min, and its color intensity gradually increased with time. We observed that the bands had no significant change after 15 min (Fig. 3D). While for the low-concentration group, it took about 20 min for the bands to become clearly visible (Fig. 3C). Thus, 20 min was finally determined as the optimal reaction time.

### The Limit of Detection Test of ERA Assays

**Fig. 4:**
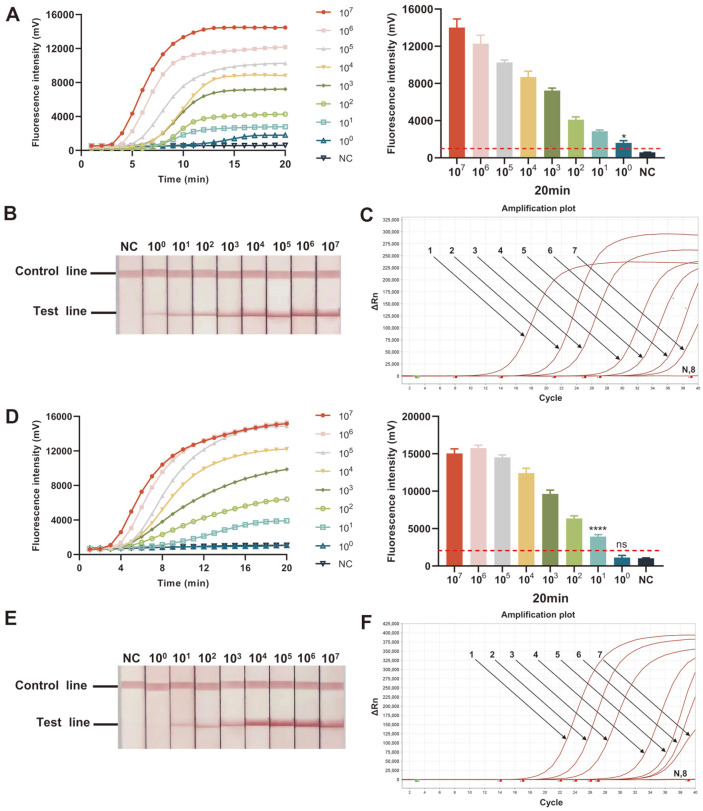
Comparison of the limit of detection (LOD) of real-time ERA, ERA-LFD, and real-time PCR assays. Fluorescence curves and final fluorescence intensities of real-time ERA performed with a serial dilution from 10^7^ to 10^0^ copies/ μl of HPV16 (**A**) and 18 (**D**) plasmids containing E6/7 genes. The threshold is illustrated as red dashed lines at 996.7 and 2053.8 for HPV16 and 18, respectively. ERA-LFD assay was conducted with a series of diluted HPV16 (**B**) and 18 plasmids (**E**) from 10^7^ to 10^0^ copies/μl. Detection of HPV16 (**C**) and 18 (**F**) using real-time PCR. 1-8: 10^7^ to 10^0^ copies/μl; N/NC: negative control; *: *p* < 0. 05; ****: *p* < 0.0001; ns: no significance.

Under the optimal reaction conditions verified before, we used various concentrations (10^7^–10^0^ copies/μl) of recombinant plasmids as templates to determine the LODs of the developed HPV16/18 ERA assays. For the identification of HPV16, the results showed that real-time ERA and ERA-LFD assays can detect as low as 10^0^ copies/μl of the target gene (Figs. 4A and 4B). While for the HPV18 testing, the LODs of the two methods could only reach 10^1^ copies/μl (Figs. 4D and 4E). The results of real-time PCR assay revealed that the LODs for the detection of HPV16 and 18 were both 10^1^ copies/μl (Figs. 4C and 4F), which correspond to that in the study of Bordigoni *et al*. [34]. These data indicated the LODs of ERA assays are comparable to or even lower than those of the real-time PCR method.

### The Specificity Test of ERA Assays

**Fig. 5:**
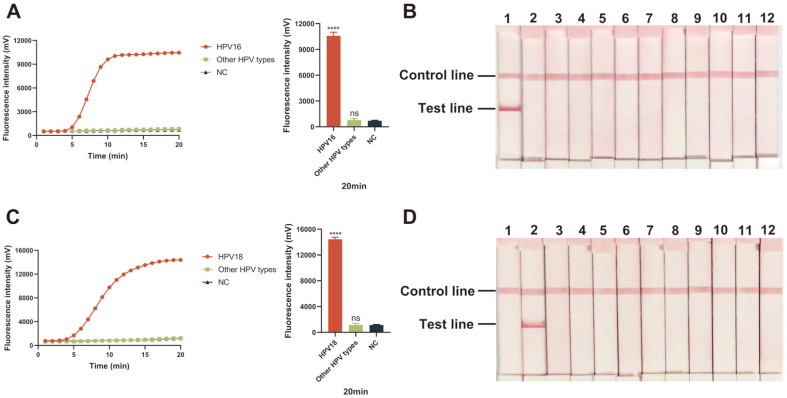
Specificity analysis of real-time ERA and ERA-LFD assays for HPV16/18 detection. Specificity analysis of HPV16 (**A**) and 18 (**C**) real-time ERA assays for the detection of HPV16, HPV18, and other 9 types of HPV by observing the fluorescence curves and final fluorescence intensities. Specificity analysis of HPV16 (**B**) and 18 (**D**) ERA-LFD assays for the detection of HPV16, HPV18, and other 9 types of HPV by observing the bands on the test lines. ****: *p* < 0.0001; ns: no significance; NC: negative control; 1-12: pHPV-16-E6/E7, pHPV-18-E6/E7, pHPV-31-E6/E7, HPV-33, HPV-35 HPV-39, pHPV-45-E6/E7 HPV-52, HPV-58, pHPV-66-E6/E7, pHPV-73-E6/E7, negative control, respectively (All the mentioned strains were shown in Table 1).

A total of 6 kinds of puc57-HPV-E6/E7 recombinant plasmids, including HPV16, 18, 31, 45, 66, and 73, were adopted for the specificity analysis of ERA assays. Other DNA templates of HR-HPVs were extracted from clinical samples validated by real-time PCR. For real-time ERA assay, except for the HPV16/18 samples, the curves of the other templates showed no amplification trend and the fluorescence value of those subtypes had no difference compared with negative control groups after the reaction (Figs. 5A and 5C). In ERA-LFD assay, no bands in reactivity with the other genotypes were present (Figs. 5B and 5D). Both the two assays exhibited high specificity.

### Comparison of the Clinical Performance of Real-Time ERA Assay, ERA-LFD, and Real-Time PCR Using Clinical Samples

We evaluated real-time ERA and ERA-LFD assays using clinical samples from 54 patients positive for HPV16/ 18 (36 HPV16 positive and 18 HPV18 positive) and 60 negative controls (14 patients positive for other HR-HPVs and 46 healthy individuals) who had been previously validated and distinguished using real-time PCR in the hospital. Among the HPV16/18 positive samples, real-time ERA assay identified 35 as positive for HPV16 and 17 for HPV18, with kappa values of 0.978 and 0.963, respectively. The kappa values between ERA-LFD and real-time PCR assays were 1.000 and 0.963 for the detection of HPV16 and HPV18, respectively (Table 3). The results indicate that the sensitivity and specificity of our developed ERA assays are comparable to real-time PCR assay, which is a gold standard assay for HPV detection, and have the potential to replace existing detection methods in the field.

## Discussion

Until now, the main approach to identifying HR-HPV types has evolved from restriction endonuclease cleavage analysis and DNA-DNA/RNA blot hybridization to PCR-based analysis [35]. During the design process of PCR primers, the highly conserved L1 gene or the E6 and E7 oncogenes were frequently chosen as target regions. However, targeting the L1 gene may cause missed diagnoses of advanced cervical cancer because the L1 gene is frequently lost during the viral DNA integration into host genomic DNA. So E6 and E7 regions have been regarded as preferable areas [36], and thus we designed and screened the specific primers or probes targeting HPV16-E6 and HPV18-E7 genes.

In this experiment, we designed three upstream and three downstream candidate primers for each HPV subtype, which are directly related to the sensitivity and specificity of ERA technique, and screened for the optimal primer combination for the following application. The length of primers for ERA assays should be greater than that of real-time PCR primers because recombinase function improves with increasing primer length. However, the risk of forming dimers and hairpins also increases; thus, we set the primer length at 30–35 bp. Besides, 5–9 mismatches in the primers can be tolerated by ERA assays, but the dimers and hairpins should be avoided [29]. To evaluate the performance of each candidate primer set, not only the quantity of ERA products, but also the start time of the amplification was considered. The real-time ERA assay is preferred over AGE to screen for the optimal primer set because it can determine the amplification efficiency of the primer sets by monitoring fluorescence signals during the entire amplification process.

In the present study, we developed real-time ERA and ERA-LFD assays as two rapid and sensitive molecular diagnostic methods for HPV16 and 18. The whole operation procedure of the two assays can be completed within 25 min at a constant temperature between 35–43°C, and the test results can be visually evaluated by the amplification curves or the bands presented on dipsticks. Both ERA assays showed high specificity and had no cross-reaction with other common HPV types. The two methods had similar LODs of 10^0^ and 10^1^ copies/μl for the detection of HPV16 and 18, respectively, which are comparable to those of the PCR-based methods [34, 37, 38]. We also evaluated the ERA assays using a total of 114 clinical samples previously validated by real-time PCR and found high consistency between the results of our developed ERA assays and those of the real-time PCR assay.

When comparing the performance of real-time ERA and ERA-LFD assays, we found that using real-time ERA has the unique advantage of reducing the false positive rate because the reaction tubes remained closed during the reaction process and aerosol contamination can thus be avoided. The results of real-time ERA assay can be presented in digital form by a portable and lightweight(<1.5 kg) fluorescence monitoring device, which has an internal battery and can last for a long time in the on-site environment without electricity. For ERA-LFD assay, the test strip is extremely portable and the isothermal condition for amplification can be easily achieved by water bath or even at physiological temperature. Both the ERA assays are easy to implement with no need for sophisticated equipment and laboratory environment, which makes them feasible to apply in resource-poor areas.

Currently, many isothermal amplification techniques have been developed and evaluated for HPV detection. Kumvongpin *et al*. developed a LAMP-LFD detection method for HPV16/18, which can detect 10^1^ copies/μl of template DNA in 45 min [39]. However the primer design for LAMP is very complex and the requirement of temperature is relatively high (around 60°C), which limits its use in low-resource settings. Ma *et al*. developed HR-HPV group detection methods by combining RPA with LFD and reverse dot blot (RDB). However, without the strict screening of primers, the LODs of their developed methods can only reach 10^2^ copies per reaction, and robustness cannot be maintained when detecting samples at low concentrations [40]. Zheng *et al*. combined RPA with CRISPR-Cas12a technology to quantitatively detect HPV16 and 18 [41]. While the CRISPR-Cas system showed high sensitivity and accuracy in the application of molecular diagnostics, the associated cost and time increases cannot be ignored. The ERA assays have the advantages of low cost, high sensitivity and specificity, which demonstrate their application potential in clinical diagnosis and laboratory research.

Our study has some limitations. First, the evaluation of the clinical performance of the ERA assays requires a larger number of clinical samples. Moreover, in this study, we used the column extraction method to acquire the DNA templates of samples. Simpler methods for nucleic acid extraction, such as nucleic acid releasing agents, have been successfully applied and show the advantages of simplifying the operation process and saving time [42, 43]. Our future work will focus on optimizing the procedure of nucleic acid extraction and rendering the entire process of detection more feasible for POCT.

In conclusion, we developed real-time ERA and ERA-LFD assays, which showed high sensitivity, robustness, and specificity for the rapid detection of HPV16 and 18 from clinical samples. The convenience and low cost of ERA assays render them suitable for application in the field or resource-limited environments. Moreover, the ERA assays have great potential for becoming model platforms for the detection of other pathogens.

## Supplemental Materials

Supplementary data for this paper are available on-line only at http://jmb.or.kr.

## Figures and Tables

**Table 1:** Strains used in this study.

Strain	Source	Reference	Method		
			Real-time ERA	ERA-LFD	Real-time PCR
pHPV-16-E6/E7	Synthesized	NCBI NC_001526.4	+	+	+
pHPV-18-E6/E7	Synthesized	NCBI GQ180792.1	+	+	+
pHPV-31-E6/E7	Synthesized	NCBI LR862053.1	-	-	-
pHPV-45-E6/E7	Synthesized	NCBI LR862061.1	-	-	-
pHPV-66-E6/E7	Synthesized	NCBI LC511686.1	-	-	-
pHPV-73-E6/E7	Synthesized	NCBI LR862011.1	-	-	-
HPV-33	Self-isolate	-	-	-	-
HPV-35	Self-isolate	-	-	-	-
HPV-39	Self-isolate	-	-	-	-
HPV-52	Self-isolate	-	-	-	-
HPV-58	Self-isolate	-	-	-	-

NCBI=National Center for Biotechnology Information; Self-isolates were extracted from clinical samples

**Table 2:** Primers and probes used in this study.

Oligo name	Sequence 5'-3'	Assays
HPV16-F	TCCAGATGTCTTTGCTTTTCTTCAGGACAC	Real-time ERA, ERA-LFD
HPV16-R	TACTGCAAGCAACAGTTACTGCGACGTGAG	Real-time ERA
HPV16-nfo-R	Biotin- TACTGCAAGCAACAGTTACTGCGACGTGAG	ERA-LFD
HPV16-exo-Probe	AACGGTTTGTTGTATTGCTGTTCTAATGT[FAM-dT] [THF] [BHQ1-dT] TCCATACAAACTAT [C3-spacer]	Real-time ERA
HPV16-nfo- Probe	[FAM]AACGGTTTGTTGTATTGCTGTTCTAATGTT[THF ]TTCCATACAAACTAT[C3-spacer]	ERA-LFD
HPV18-F	TTCGGCTCGTCGGGCTGGTAAATGTTGATG	Real-time ERA, ERA-LFD
HPV18-R	CCCCAAAATGAAATTCCGGTTGACCTTCTA	Real-time ERA
HPV18-nfo-R	Biotin- CCCCAAAATGAAATTCCGGTTGACCTTCTA	ERA-LFD
HPV18-exo-Probe	CTCCATCTATTTCATCGTTTTCTTCCTC[FAM-dT] G[THF]G[BHQ1-dT] CGCTTAATTGCTC[C3-spacer]	Real-time ERA
HPV18-nfo- Probe	[FAM]CTCCATCTATTTCATCGTTTTCTTCCTCTG[THF] GTCGCTTAATTGCTC[C3-spacer]	ERA-LFD
HPV16-PCR-F	AATGTTTCAGGACCCACAGG	Real-time PCR [34]
HPV16-PCR-R	GTTGCTTGCAGTACACACATTC	Real-time PCR
HPV18-PCR-F	AATTCCGGTTGACCTTCTATGT	Real-time PCR [34]
HPV18-PCR-R	GGCTGGTAAATGTTGATGAT	Real-time PCR

F, forward primer; R, reverse primer, FAM, 6-carboxyfuorescein; THF, tetrahydrofuran; BHQ, black hole quencher; C3-Spacer, 3' phosphate blocker

**Table 3:** The detailed comparison of three methods for the detection of HPV16 and 18 in 114 clinical samples.

Assay			Real-time PCR			Sensitivity (%)	Specificity (%)	PPV (%)	NPV (%)	P	K
			Positive	Negative	Total						
Real-time ERA assay	HPV 16	Positive	35	0	35	97.22	100	100	98.36	<0.001	0.978
		Negative	1	60	61						
		Total	36	60	96						
	HPV 18	Positive	17	0	17	94.44	100	100	98.36	<0.001	0.963
		Negative	1	60	61						
		Total	18	60	78						
ERA-LFD assay	HPV 16	Positive	36	0	36	100	100	100	100	<0.001	1.000
		Negative	0	60	60						
		Total	36	60	96						
	HPV 18	Positive	17	0	17	94.44	100	100	98.36	<0.001	0.963
		Negative	1	60	61						
		Total	18	60	78						

PPV, Positive Predictive Value; NPV, Negative Predictive Value; *P*, P value; *K*, Kappa value
