# Supplementary material for: Development of Enzymatic Recombinase Amplification Assays for the Rapid Visual Detection of HPV16/18
Source: J Microbiol Biotechnol. 2023 May 19;33(8):1091–100. doi: 10.4014/jmb.2304.04009 (PMC10468672; doi:10.4014/jmb.2304.04009)
Supplement: Supplementary file 1 [file jmb-33-8-1091-supple.pdf]

J. Microbiol. Biotechnol.
https://doi.org/10.4014/jmb.2304.04009

Supplementary Table

Table S1. The sequences of candidate primers.

Name	Sequence (5'-3')	Targeted gene
HPV16-F1	CATTTACCAGCCCGACGAGCCGAACCACAA	HPV16-E6
HPV16-F2	TCCAGATGTCTTTGCTTTTCTTCAGGACAC	
HPV16-F3	ACAGTGGCTTTTGACAGTTAATACACCTAA	
HPV16-R1	GCAAGCAACAGTTACTGCGACGTGAGGTAT	
HPV16-R2	TACTGCAAGCAACAGTTACTGCGACGTGAG	
HPV16-R3	ATGCACAGAGCTGCAAACAACTATACATGA	
HPV18-F1	GCTCGAAGGTCGTCTGCTGAGCTTTCTACT	HPV18-E7
HPV18-F2	TTGTGGTTCGGCTCGTCGGGCTGGTAAATG	
HPV18-F3	TTCGGCTCGTCGGGCTGGTAAATGTTGATG	
HPV18-R1	CCCCAAAATGAAATTCCGGTTGACCTTCTA	
HPV18-R2	AGAGCCCCAAAATGAAATTCCGGTTGACCT	
HPV18-R3	TTAGAGCCCCAAAATGAAATTCCGGTTGAC	
